# Dynamic beam control based on electrically switchable nanogratings from conducting polymers

**DOI:** 10.1515/nanoph-2022-0801

**Published:** 2023-03-03

**Authors:** Yohan Lee, Julian Karst, Monika Ubl, Mario Hentschel, Harald Giessen

**Affiliations:** 4th Physics Institute and Research Center SCoPE, University of Stuttgart, Pfaffenwaldring 57, 70569 Stuttgart, Germany

**Keywords:** beam diffraction, electrically switchable, nanogratings, nanooptics, plasmonics

## Abstract

Surging interests in point-of-device miniaturization have led to the development of metasurface-based optical components. Here, we demonstrate an electrically-driven ultracompact beam controller in the infrared spectral range. The effect benefits from diffraction gratings consisting of the commercially available conductive polymer PEDOT:PSS, which exhibits metal-to-insulator transition characteristics upon electrical biasing. By combining several metagratings with different superlattice periods in electrically isolated areas, our device enables diffraction beams at 16 and 33.5° when applying voltages of only ±1 V. Furthermore, no diffraction is realized by switching off the plasmonic property of the gratings. Dynamic control of electromagnetic wave via the presented platforms could be transformative for sensing, imaging, and communication applications.

## 1 Introduction

Over the past decades, metasurfaces for the passive manipulation of the spatial and spectral properties of electromagnetic waves have been studied as a key component in terms of device miniaturization [1–3]. Metasurfaces enable controlling the wavefront of the scattered light, thereby demonstrating a number of low-profile optical components for polarization control and detection [4–6], holograms [7–9], or focusing [10–12]. Beyond fundamental limits of passive type platforms, the focus of research is shifting towards dynamically tunable metasurfaces, which offer diverse applications and even replace conventional bulky optical elements [13–15]. In general, such miniaturized optical platforms have been realized by incorporating active materials, of which dielectric permittivity can be dynamically controlled via the application of an external stimulus, such as mechanical actuation [16–22], electrical bias [23–35], laser pulse [36–38] or heat treatment [39, 40].

Among various functionalities that active metasurfaces can implement, dynamic beam steering has been considered one of the most crucial technologies because it can offer diverse applications that directly relate to commercial products in light detection and ranging (LiDAR) devices [41, 42], wireless communication [43–45], and high-resolution imaging [46]. Recently, various kinds of ultracompact dynamic beam steering and switching devices have been demonstrated by combining metasurfaces with diverse active materials such as micromechanical systems (MEMS) [16–18], transparent conducting oxides (TCOs) [23–28], liquid crystal [32, 33], or phase-change materials [34–37]. However, they have still suffered from low efficiency, small scanning angle, or high energy consumption. In this work, we put forward an electrically switchable metagrating, a one-dimensional metasurface, which enables actively controllable beam steering by diffraction of scattered electromagnetic waves in the infrared wavelength range. To achieve dynamic tunablity, we employ PEDOT:PSS (poly(3,4-ethylenedioxythiophene):poly-styrene sulfonate) as an active material, whose dielectric function can be changed upon an applied voltage in the range between only +1 V and −1 V [47, 48]. In particular, PEDOT:PSS is employed as a phase change material, which undergoes a reversible metal-to-insulator transition [47–52].

## 2 Results

**Figure 1:**
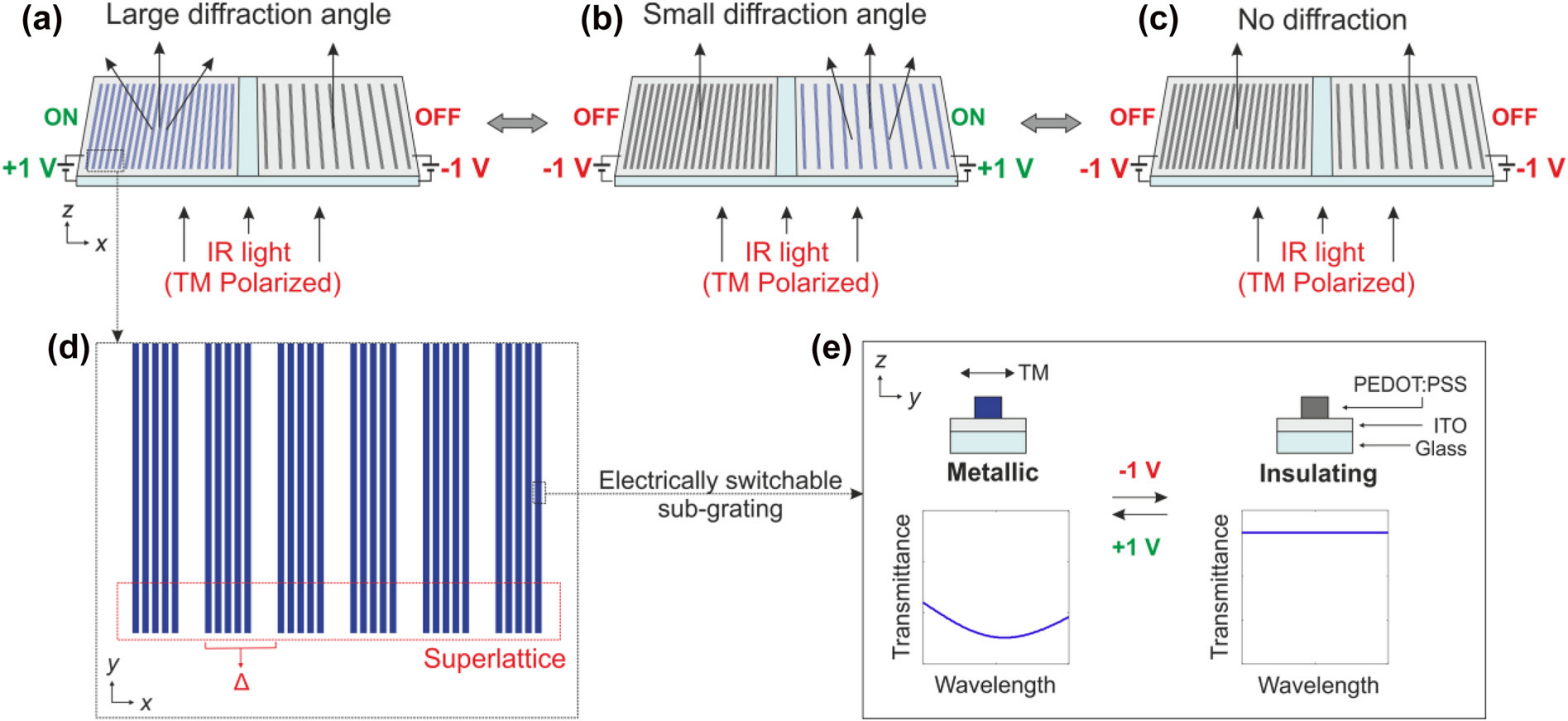
Concept of the beam deflector from electrically switchable metagratings. (a–c) Schematic illustration of a beam controller consisting of two electrically switchable metagratings. The metagratings are electrically insulated and independently controlled by applying voltages. An applied voltage of +1 V or −1 V turns the power of diffracted components by a grating ON or OFF, respectively. Three cases are demonstrated depending on the individual voltage applied to the metagratings. (a) Diffraction beam with large angle (left: ON, right: OFF), (b) diffraction beam with small angle (left: OFF, right: ON), (c) no diffraction beam (left: OFF, right: OFF), (d) each metagrating has a different superlattice period, which determines the angle of diffraction beam. The gratings are composed of electrically switchable plasmonic sub-gratings, which feature lithographically structured PEDOT:PSS nanowires (width: 270 nm, period: 600 nm, height: 90 nm). The sub-grating exhibits a localized surface plasmon resonance for incident TM-polarized light. This makes the interaction of light with the switchable metallic grating very efficient. (e) Metal-to-insulator transition of the polymer depending on applying voltages is a key mechanism of the device. Left: in metallic polymer state with an applied voltage of +1 V, the plasmonic resonance turns on. Right: on the other hand, the plasmonic resonance is turned off when applying a voltage of −1 V, which switches the polymer into insulating state.

The concept of our beam controller is depicted in Figure 1. The device consists of two electrically switchable metagratings, which are placed on electrically separated areas to be independently controlled (see Figure 1a). The two metagrating have a different superlattice period in order to obtain two different diffraction angles. The metagrating on the left side possesses a shorter period (larger diffraction angle) compared to that on the right side. With this combination, three different states of beam diffraction are realized depending on how the voltages are applied to each grating (illustrated in Figure 1a–c). In Figure 1a, the metagrating on the left side is turned ON (displayed in blue) by an applied voltage of +1 V, which means it becomes optically metallic. Simultaneously, we turn the metagrating on the right side OFF (displayed in gray) by applying a voltage of −1 V to make it optically insulating. In this situation, the incoming infrared (IR) light interacts efficiently with only the metallic grating resulting in the generation of the diffracted beams, while passing through the insulating grating without much interaction. In contrast, in Figure 1b, voltages of −1 V and +1 V is applied to the left and right areas, respectively. The diffraction occurs by the metagrating on the right side leading to a small diffraction angle. Furthermore, no diffraction is realized by switching both gratings fully OFF with applied voltages of −1 V (see Figure 1c). The metagrating has a superlattice period (Δ) since it is composed of five sub-gratings (Figure 1d). This period determines the angle of the diffraction beam. The diffraction angle *θ* by gratings as a function of the superlattic period Δ is given as

(1) $$\theta =\sin^{-1}\frac{m\lambda }{\Delta }$$

where *λ* denotes the incident wavelength and *m* is the order of diffraction. In this work, only the zeroth- and first-order diffraction components are considered. Based on this equation, the fact that the angle of the diffraction generated by the left metagrating is larger can be explained because its superlattice period is smaller.

For wavelengths roughly longer than 2 µm, the optical property of this metallic polymer can be reversibly switched from metallic to insulating by its electrochemical reaction states [48] (see Figure S1 in the Supplementary Information). The sub-grating is designed in such way to exhibit a plasmonic resonance at the operating wavelength by tailoring its geometric parameters, in particular width and height of the PEDOT:PSS wires. The PEDOT:PSS sub-gratings placed on ITO (indium-tin-oxide)-coated glass substrates show optically metallic properties in the pristine state. When a voltage of −1 V is applied, the material will be electrochemically reduced via the surrounding electrolyte and becomes optically insulating, thus most incident waves are transmitted without undergoing absorption or reflection. Its metallic state can be recovered by being electrochemically oxidized with an applied voltage of +1 V. One can observe, the plasmonic resonance in transmittance resulting from the interaction between the metallic material and incident light.

**Figure 2:**
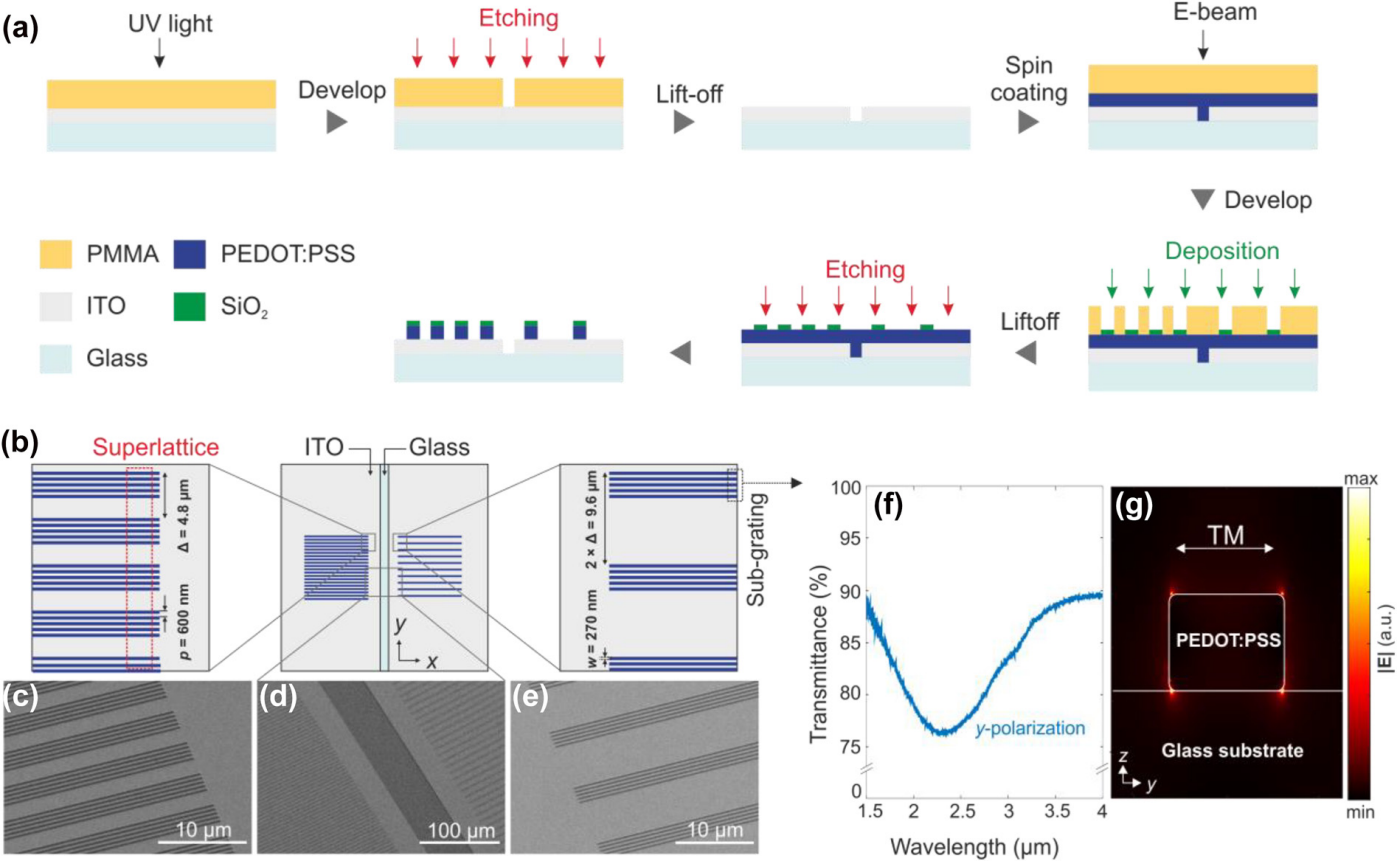
Sample design and fabrication of a beam deflector based on metallic polymer metagratings. (a) Fabrication process for the beam controller consisting of two electrically insulated metagratings. (b) Schematic of the switching device. The metagrating on the left side has a superlattice period of 4.8 µm, while the metagrating on the right side has 9.6 µm. Both feature identical sub-gratings with the same width (270 nm) and period (600 nm). The height of the PEDOT:PSS wires is 90 nm. (c–e) SEM images of three parts in (b). (f) Measured transmittance spectrum of a sub-grating of metallic polymer. A plasmonic resonance is observed around the wavelength of 2.3 µm with *y*-polarized (TM-polarized) incidence. (g) Field profile for plasmonic resonance at the resonant wavelength.

The fabrication process is described in Figure 2a: ITO-coated glass (ITO thickness is 20 nm) is prepared as a substrate to be used as a working electrode in electrochemical reaction. A resist (AZ5214E) is spin-coated on the substrate, baked (90 °C, 1 min), and exposed by ultraviolet light. The sample is soaked in developer (AZ-developer mixed with water [1:1]), and etched for electrical separation of the ITO patches. After removing residuals by acetone, a 90 nm of PEDOT:PSS layer (Heraeus PH1000, Ossila) is spin-coated and dried (120 °C, 15 min). The film is covered with a double-layered poly(methyl-methacrylate) (PMMA) resist (Allrestist AR-P 642.06 200k, Allresist AR-P 672.02 950k), and exposed by an electron-beam (Raith eLine Plus). After development in methylisobutylketone (MIBK), 30 nm silicon-dioxide (SiO_2_) is deposited as a hard-mask by an electro-gun evaporator. After lift-off, PEDOT:PSS metagrating is structured by argon (Ar) ion-beam etching.

The schematic of the fabricated structure is depicted in Figure 2b. As mentioned, the ITO layer on the glass substrate is divided into two areas with 5 µm gap width. On each area, metallic polymer metagratings (size: 100 × 100 µm^2^) are placed to be independently controlled by applied voltages. Note that the metagratings have different superlattice periods (left side: 4.8 µm, right side: 9.6 µm). This makes it possible to implement two different diffraction angles with one platform, depending on ON- and OFF-states of the metagratings. Scanning electron microscope (SEM) images taken of three magnified areas (the individual metagratings on the both sides and the etched region at the center of the sample) show excellent fabrication quality (see Figure 2c–e). The sub-gratings as building blocks for both metagratings have the same parameters: 270 nm of nanowire width and 600 nm of period. The parameters of the sub-gratings are chosen to lead to a plasmonic resonance at the target wavelength of 2.65 µm. This wavelength is chosen to avoid strong absorption by the material itself. When measuring the spectral response of the PEDOT:PSS grating, its transmittance spectrum of the dry state exhibits a resonance dip around ∼2.35 µm. This result shows a good agreement with numerical calculation data (see Figure S2 in the Supplementary Information). The plasmon resonance shifts slightly red near the target wavelength, when the nanostructured pattern is immersed in liquid electrolyte (acetonitrile) for the electrochemical experiment. On the basis of the measured dielectric function of PEDOT:PSS, we perform the full-wave electromagnetic simulations to check the characteristics of the plasmonic resonance. In Figure 2g, the optical response of the PEDOT:PSS sub-grating unit cell around the resonant wavelength is characterized at normal incidence under TM-polarization. The electric field enhancement increases drastically with maximum values near the nanowire corners, which is the characteristics of a localized surface plasmon resonance.

**Figure 3:**
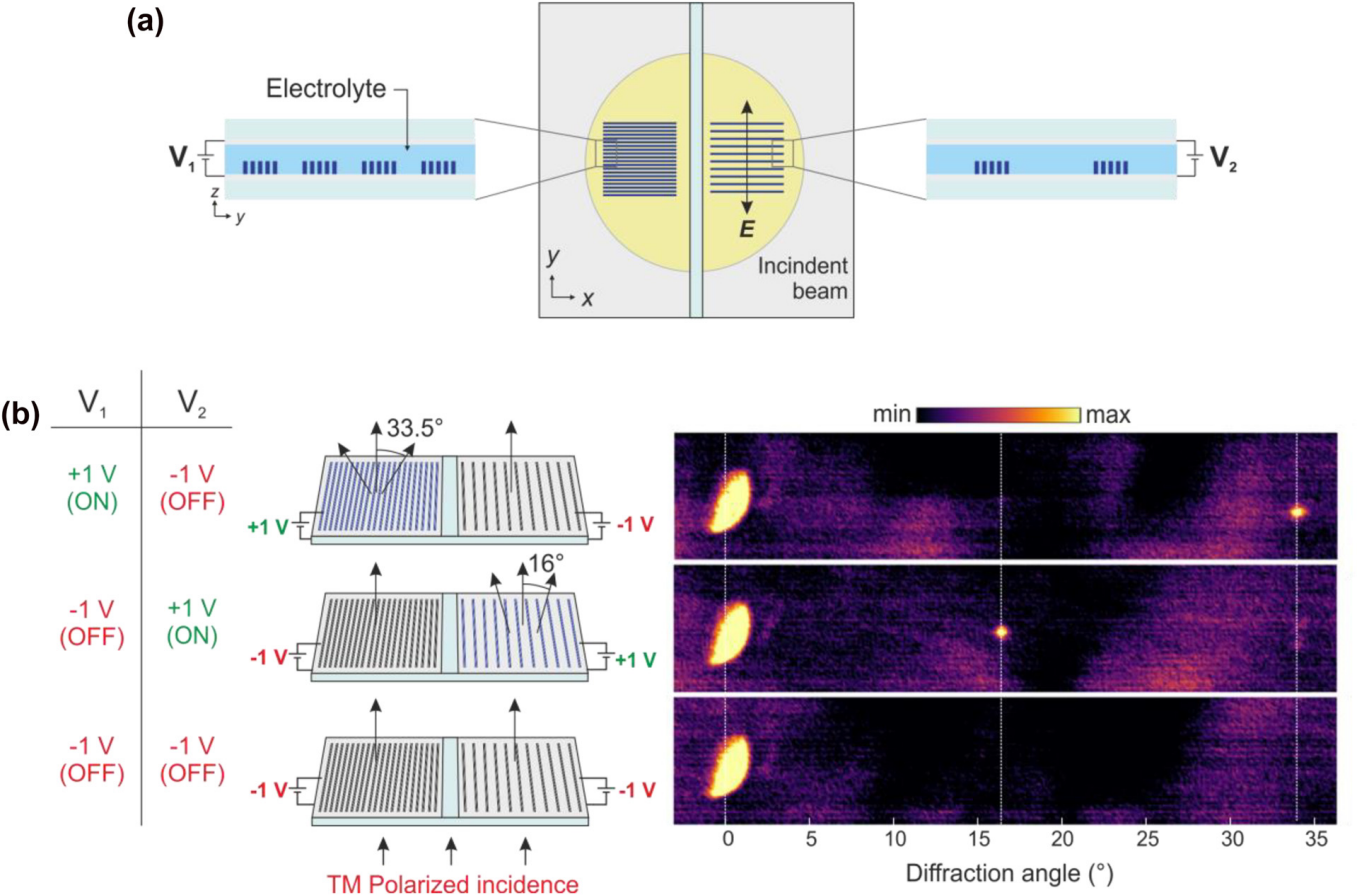
Experimental results of a beam deflector based on metallic polymer metagratings. (a) Schematic of the sample for dynamic diffraction measurement. Polymer metagratings on ITO-coated glass are covered with an electrolyte for an electrochemical switching of the polymer, followed by another ITO-coated glass as counter electrode. The beam size of incidence is large enough to cover both metagratings. The state of the metagrating on the left side is controlled by V_1_, while V_2_ controls the metagrating on the right side. (b) Three different states categorized by applied voltages, their schematics, and experimental results. Top: 33.5° of diffraction angle (V_1_: ON, V_2_: OFF), middle: 16° of diffraction angle (V_1_: OFF, V_2_: ON), bottom: no diffraction (V_1_: OFF, V_2_: OFF). The experimental results are plotted with the color axis using saturated maximum and minimum values (the unsaturated plot is provided in Figure S3 in the Supplementary Information).

The control of diffraction beam by electrical switching is carried out with a two-electrode cell (Figure 3a). A flipped ITO-coated glass serves as a counter electrode. We put SiO_2_ spheres (diameter: 10 µm) around the pattern to secure enough space between two electrodes and protect the nanostructures from a physical damage. The electrochemical cell is filled with the electrolyte (0.1 mol/L TBAPF_6_ in acetonitrile). To be switchable separately, each side (working electrode 1 and 2) is connected to the voltage of V_1_ and V_2_ (the potential difference between working and counter electrodes), respectively. Note that the size of the incident beam is large enough to cover both metagratings. The polarization of incidence is perpendicular to the direction of the long-axis of the grating in order to exploit the plamonic resonance. Our diffraction beam controller demonstrates three different states. The results are shown in Figure 3b (experimental setup is provided in Figure S4 in the Supplementary Information). In the first state (top), the metagrating on the left side is turned ON (+1 V) whereas the metagrating on the right side is turned OFF (−1 V). Only the metagrating on the left side generates the diffracted components upon the linearly polarized TM wave and the angle of the first-order diffraction beam is about 33.5° according to the Equation (1). Plotting the measured diffraction beam that was recorded with a Spiricon IR camera, we find the beam at 0° to give the zeroth order diffraction component, which is always there regardless of the state. The size of the diffracted beam is smaller than that of the zeroth order beam because only a part of light is interacting with the nanopattern that can cause diffraction. The second state (left side: OFF, right side: ON) shows a different angle of diffraction compared to the first state (middle of Figure 3b). We observe the first-order diffraction beam at 16°. Furthermore, both metagratings are set OFF and turn into the insulating state by an applied voltage of −1 V to both sides (bottom of Figure 3b). The diffracted power of both metagratings is switched OFF and no diffraction is observed. The diffraction efficiencies of the +1st-order beam by our two different metagratings in Figure 3b (defined as the power of the first-order diffraction beam divided by the power summation of the 0th- and the +1st-order beam) are 1.11% and 1.21%, respectively (The calculated power of each diffracted beam is provided in Figure S5 in the Supplementary Information), and it is confirmed by the measurement (see Figure S6 in the Supplementary Information). In case of following a different kind of definition of the diffraction efficiencies (the ratio between the power of diffracted beam and the power of input), those of the +1st-order beam would be 0.0076% and 0.0109%, respectively. They are limited right now by several factors: (I) the value of the dielectric function of the PEDOT:PSS at the incident wavelength, (II) the filling factor (duty cycle) of our structure, and (III) the thickness of our polymer nanowires. In this work, the diffraction efficiency is adjustable by tailoring the number of sub-gratings, which is directly related to the factor (II) (see Figure S7 in the Supplementary Information). Considering that the ON–OFF switching speed of the PEDOT:PSS nanoantennas of 90 nm thickness exhibit up to 30 Hz of switching frequencies [48], our devices are expected to show similar.

## 3 Conclusions

In summary, we have demonstrated a dynamic diffraction beam controller based on electrically switchable metallic polymer metagrating. The varying angle of the first-order diffraction is achieved via two independently controllable one-dimensional PEDOT:PSS gratings. The diffraction of the metagrating can be switched ON and OFF by applying voltages of +1 V and −1 V, respectively. Using this concept, three different states of beam deflection with angles of up to 33.5°. We expect the performance and functionality of beam steering can be improved by using a phase-tunable grating or through combination with materials such as metals that can strongly confine the electromagnetic field. This approach can improve the functionalities of PEDOT:PSS-based metasurfaces because it enables unidirectional deflection, as well as suppression of non-diffracted components which are unnecessary. Development in materials science could in the future improve the carrier density and hence the diffraction efficiency. Furthermore, it could shift the plasma frequency to higher frequencies and allow for operation at telecom wavelength. Additionally, recent reports of using gel or solid electrolytes in combination with encapsulation might make the device very compact and standalone, also achieving switching rates in the tens of Hertz range [52]. These features may be very useful for demonstration of future electrically tunable optical components such as beam steering devices, dynamic holograms, and ultracompact lenses with reconfigurable focal lengths.

## 4 Materials and methods

### 4.1 Electrochemical switching for beam steering

A home-built electrochemical (EC) cell with optical access is used to electrically switch the metallic polymer metagratings. We use a two-electrode setup, where the voltage is controlled via a potentiostat (BioLogic SP-200). The ITO-coated glass substrate where the metallic polymer patterns are placed serves as the working electrode. The counter electrode (flipped ITO-coated glass) is placed above nanopattrens. The EC cell is filled with an electrolyte (0.1 mol/L TBAPF_6_ in Acetonitrile).

### 4.2 Spectral IR measurements

We use a Fourier-transform infrared (FTIR) spectro-microscopy setup (Bruker Vertex 80 spectrometer with Hyperion 3000 microscope) to measure the transmittance spectrum of the metallic polymer nanograting. ITO-covered glass substrates are used as reference.

## Supplementary Material

Supplementary Material Details
