# Supplementary material for: Dynamic beam control based on electrically switchable nanogratings from conducting polymers
Source: Nanophotonics. 2023 Mar 3;12(14):2865–71. doi: 10.1515/nanoph-2022-0801 (PMC11501603; doi:10.1515/nanoph-2022-0801)
Supplement: Supplementary file 1 — Supplementary Material Details [file j_nanoph-2022-0801_suppl.docx]

**Supporting Information**

**for**

**Dynamic beam control based on electrically switchable nanogratings from conducting polymers**

*Yohan Lee^1^, Julian Karst^1^, Monika Ubl^1^, Mario Hentschel^1^, and Harald Giessen^1^**

^1^ 4th Physics Institute and Research Center SCoPE, University of Stuttgart, Pfaffenwaldring 57, 70569 Stuttgart, Germany

*Corresponding author, e-mail: giessen@physik.uni-stuttgart.de

**
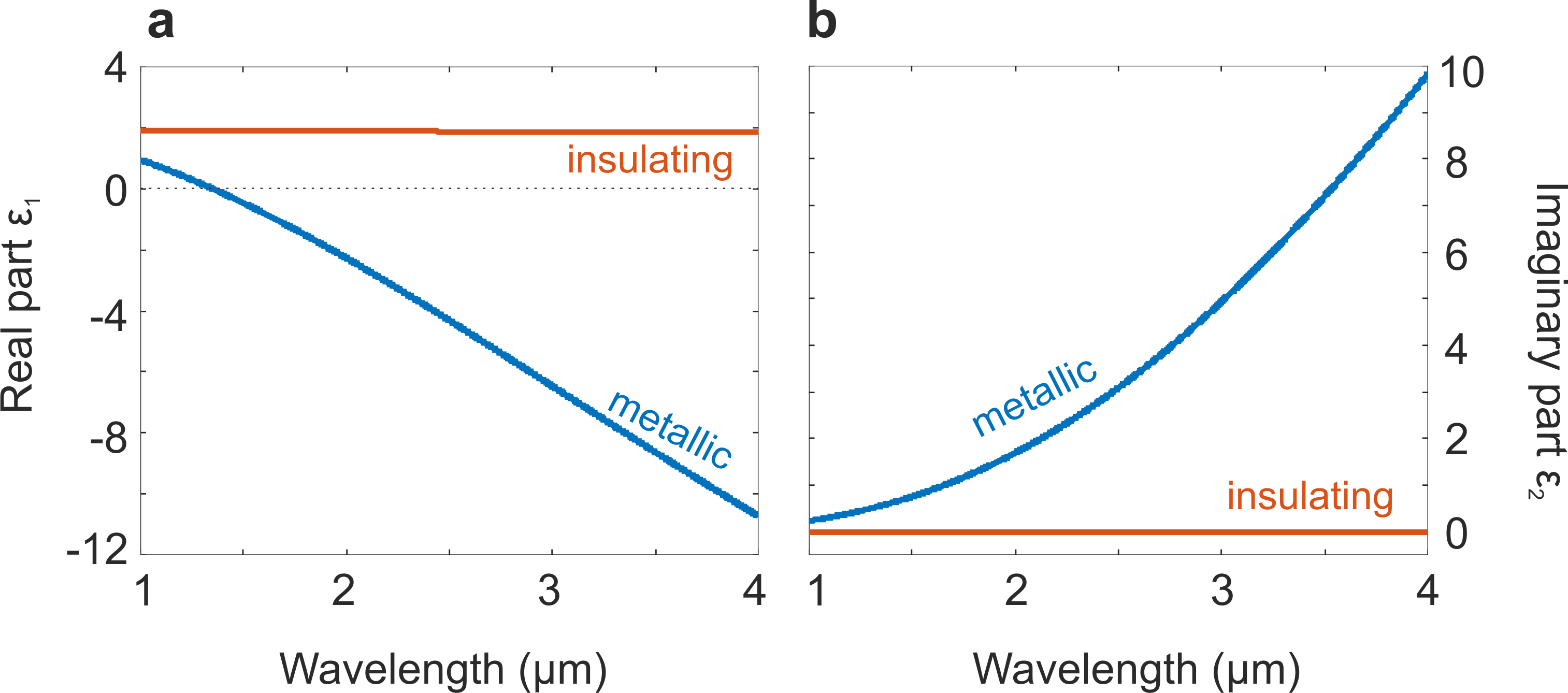
**

**Figure S1. Dielectric function of PEDOT:PSS.** (a) Real part ε_1_ of PEDOT:PSS in the metallic state (blue) and insulating state (red). The crossing where ε = 0 in the metallic state is marked. (b) Imaginary part ε_2_ of PEDOT:PSS in the metallic (blue) and insulating state (red).

**
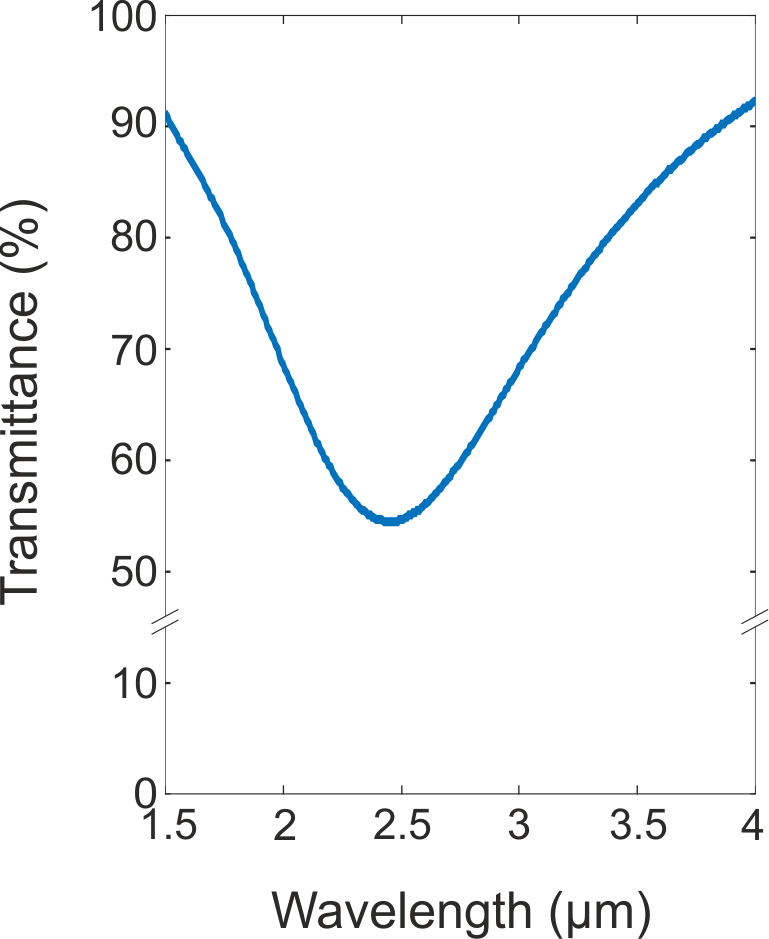
**

**Figure S2. Simulation data for plasmonic resonance.** Simulated transmittance as function of wavelength for TM polarized light. The period of the PEDOT:PSS nanogratings is 600 nm, the width is 270 nm, and the height is 90 nm. The simulation data is obtained via Rigorous coupled wave analysis (RCWA).

**
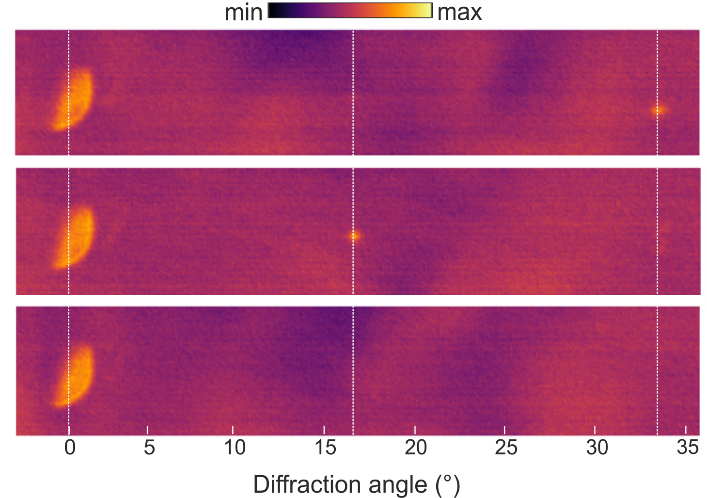
**

**Figure S3. Multi-angle beam deflector with unsaturated color scale.** The plots are the same as the results shown in Figure 3b of the main manuscript except the range of the color axis is changed. Here, the maximum and minimum values are chosen to prevent saturation of the color axis.

**
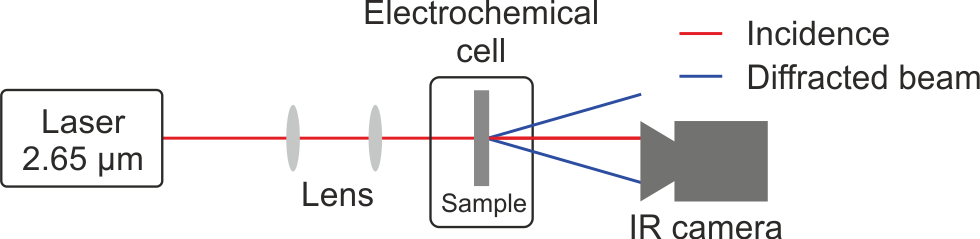
**

**Figure S4. Experimental setup to measure the diffraction by the electrically switchable metallic polymer nanogratings.** In this measurement, a tunable parametric frequency converter system (Alpha-HP, Stuttgart Instruments GmbH) for the laser illumination tuned to *λ* = 2.65 µm. A linear polarizer is used to obtain transverse magnetic (TM) polarized light. We illuminate with a collimated beam, where the beam waist is adjusted with a lens system in front of the sample. The PEDOT:PSS metagrating is placed inside the electrochemical cell to switch ON and OFF the diffraction beams by applying voltages. The beams are detected and imaged using an IR camera (Spricion Pyrocam III).


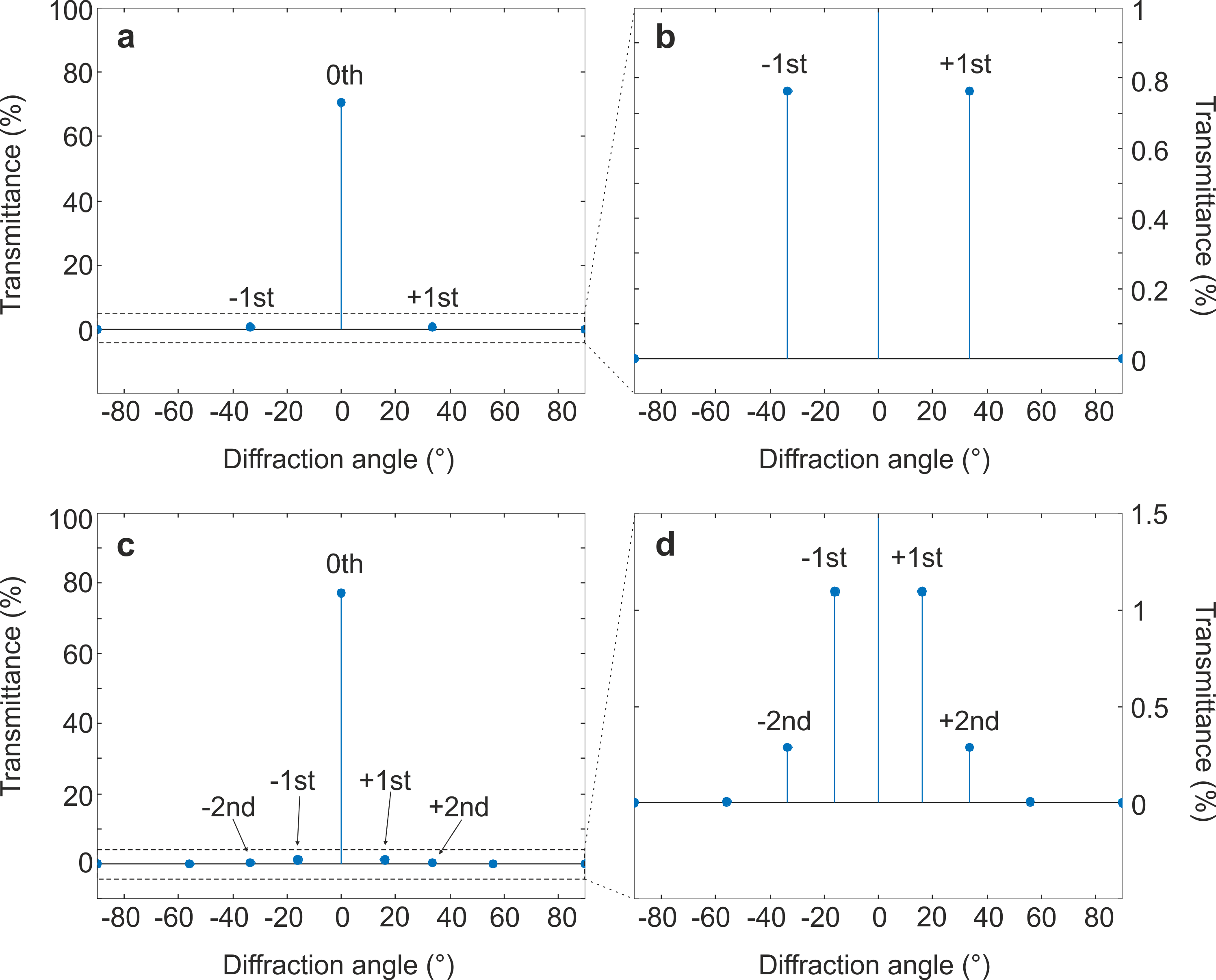


**Figure S5. Simulated transmittance of the diffraction beams of metallic polymer nanogratings.** (a) Simulated transmittance of PEDOT:PSS nanograting with superlattice period of 4.8 µm as a function of diffraction angle (TM polarized light). (b) The magnified plot of (a). (c) Simulated transmittance of PEDOT:PSS nanograting which superlattice period of 9.6 µm as a function of diffraction angle (TM polarized light). (d) The magnified plot of (c). The simulations are obtained via Rigorous coupled wave analysis (RCWA).

**
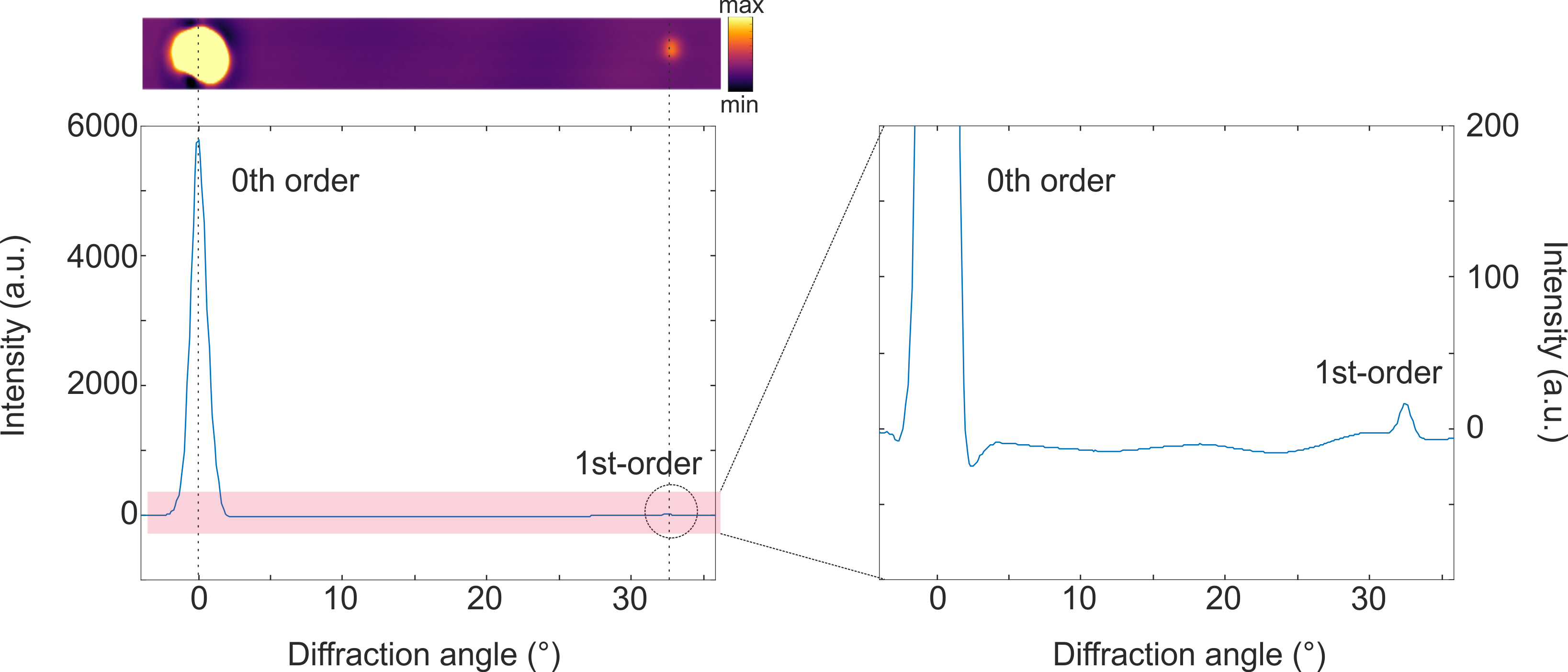
**

**Figure S6. Experimental data on the diffraction efficiency of the first order in our PEDOT:PSS grating with superperiod of 4.8 µm.** 2D image (top) of the measured diffraction beams and its intensity profile (bottom, sum along y-direction). For better visibility of the diffracted intensity, the intensity profile is magnified on the right.

**
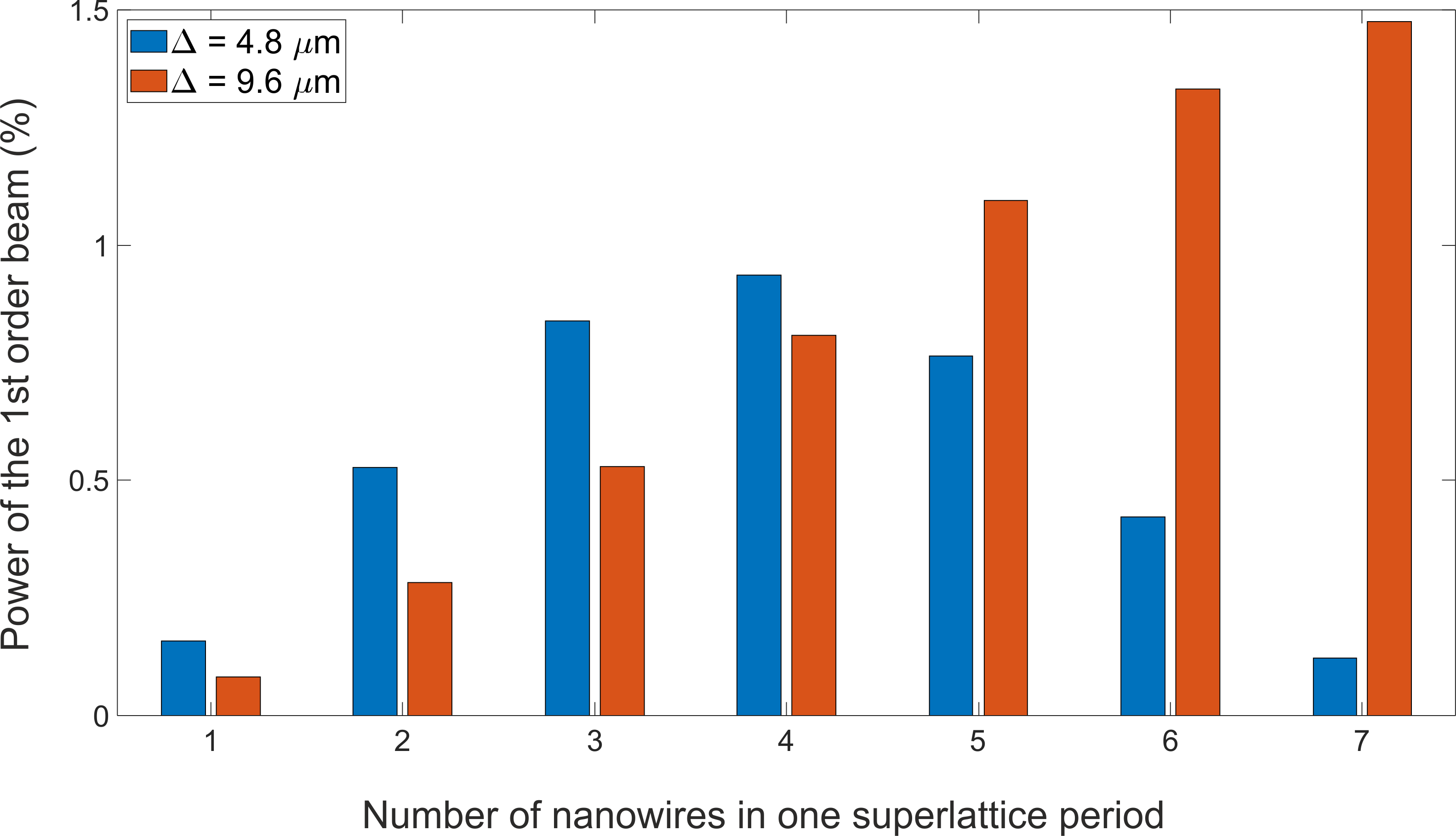
**

**Figure S7. Power of the first-order diffraction beam for our metagratings depending on the number of PEDOT:PSS nanowires within one superlattice period.** The power of diffraction beam is affected by the filling factor (duty cycle) of periodic structures. In this work, the metagrating whose superlattice period is 4.8 µm (on the left side in Figure 3a of main manuscript) can have one to seven nanowires in one superlattice period, because the period of sub-grating structure is fixed to 600 nm. On the other hand, the metagrating whose superlattice period is 9.6 µm (on the right side in Figure 3a of the main manuscript) can have one to thirteen nanowires since the superlattice period becomes doubled. The number of nanowires within one superlattice period is chosen to be 5 so that the diffraction efficiencies of both metagratings are maximized at the same time.
